# Prognostic value of Glypican family genes in early-stage pancreatic ductal adenocarcinoma after pancreaticoduodenectomy and possible mechanisms

**DOI:** 10.1186/s12876-020-01560-0

**Published:** 2020-12-10

**Authors:** Jun-Qi Liu, Xi-Wen Liao, Xiang-Kun Wang, Cheng-Kun Yang, Xin Zhou, Zheng-Qian Liu, Quan-Fa Han, Tian-Hao Fu, Guang-Zhi Zhu, Chuang-Ye Han, Hao Su, Jian-Lu Huang, Guo-Tian Ruan, Ling Yan, Xin-Ping Ye, Tao Peng

**Affiliations:** 1 Department of Hepatobiliary Surgery, The First Affiliated Hospital of Guangxi Medical University, Shuang Yong Rd. 6#, Nanning, 530021 Guangxi Zhuang Autonomous Region People’s Republic of China; 2 Department of Hepatobiliary Surgery, The Third Affiliated Hospital of Guangxi Medical University, Nanning, 530031 Guangxi Zhuang Autonomous Region People’s Republic of China; 3 Department of Colorectal and Anal Surgery, The First Affiliated Hospital of Guangxi Medical University, Nanning, 530021 Guangxi Zhuang Autonomous Region People’s Republic of China

**Keywords:** GPC family genes, Pancreatic ductal adenocarcinoma, Prognostic indicator, Mechanism

## Abstract

**Background:**

This study explored the prognostic significance of Glypican (GPC) family genes in patients with pancreatic ductal adenocarcinoma (PDAC) after pancreaticoduodenectomy using data from The Cancer Genome Atlas (TCGA) and Gene Expression Omnibus (GEO).

**Methods:**

A total of 112 PDAC patients from TCGA and 48 patients from GEO were included in the analysis. The relationship between overall survival and the expression of GPC family genes as well as basic clinical characteristics was analyzed using the Kaplan-Meier method with the log-rank test. Joint effects survival analysis was performed to further examine the relationship between GPC genes and prognosis. A prognosis nomogram was established based on clinical characteristics and prognosis-related genes. Prognosis-related genes were investigated by genome-wide co-expression analysis and gene set enrichment analysis (GSEA) was carried out to identify potential mechanisms of these genes affecting prognosis.

**Results:**

In TCGA database, high expression of *GPC2*, *GPC3*, and *GPC5* was significantly associated with favorable survival (log-rank *P* = 0.031, 0.021, and 0.028, respectively; adjusted *P* value = 0.005, 0.022, and 0.020, respectively), and joint effects analysis of these genes was effective for prognosis prediction. The prognosis nomogram was applied to predict the survival probability using the total scores calculated. Genome-wide co-expression and GSEA analysis suggested that the *GPC2* may affect prognosis through sequence-specific DNA binding, protein transport, cell differentiation and oncogenic signatures (KRAS, RAF, STK33, and VEGFA). *GPC3* may be related to cell adhesion, angiogenesis, inflammatory response, signaling pathways like Ras, Rap1, PI3K-Akt, chemokine, GPCR, and signatures like cyclin D1, p53, PTEN. *GPC5* may be involved in transcription factor complex, TFRC1, oncogenic signatures (HOXA9 and BMI1), gene methylation, phospholipid metabolic process, glycerophospholipid metabolism, cell cycle, and EGFR pathway.

**Conclusion:**

*GPC2*, *GPC3*, and *GPC5* expression may serve as prognostic indicators in PDAC, and combination of these genes showed a higher efficiency for prognosis prediction.

## Background

Pancreatic cancer (PC) is related to an unfavorable prognosis, and its mortality rate is close to its incidence rate [1]. The incidence of PC is predicted to rise 40% in the next 10 years in North America and Europe [2], and according to the latest statistics, PC ranks fourth among cancers directly causing death for men and women in the United States [3], moreover, by 2030, its rank may increase to second [4]. In China, the prognostic status of PC patients is also severe, and 5-year survival rate of patients with PC after age standardization is approximately 11.7% [5]. Due to the unique biological behaviors of PC, metastasis is present when patients are diagnosed and only 9.7% patients can be diagnosed at an early stage [6]. Furthermore, the 5-year survival rate is 9% for PC at all stages and 3% at advanced stages [3]. So far, surgical resection remains the best therapy for PC at the early stage [7]. Therefore, identifying reliable early molecular markers to improve prognosis of PC is important.

Glypican (GPC) family genes include six members (*GPC1, GPC2, GPC3, GPC4, GPC5, GPC6*), and all of the GPC family are expressed in human [8]. Glypicans are attached to the cell membrane and function in biological processes such as cell and tissue growth, embryo development, and cell movement [9, 10]. They are reported to be related to multiple diseases including various cancers. *GPC1* is upregulated in pancreatic cancer [11], esophageal cancer [12], and prostate cancer [13]. Li et al. report that *GPC1* contributes to the proliferation and motility of esophageal cancer cells through the PTEN/Akt/β-catenin pathway [14]. Increased level of GPC3 in serum could serve as a marker for hepatoblastoma [15] as well as hepatocellular carcinoma (HCC) [16, 17]. *GPC3* deletion mutation can help in diagnosis of Simpson-Golabi-Behmel syndrome type 1 (SGBS1), which is a serious genetic disease [18, 19]. Overexpression of *GPC5* may accelerate tumor progression of lymphoma [20]. In addition, *GPC5* may play a role in strengthening the interaction between Patched 1 and Hedgehog signaling in rhabdomyosarcoma [21]. *GPC5* may serve as a key gene affecting the cell cycle of podocytes in kidneys, finally causing nephrotic syndrome [22].

Pancreatic ductal adenocarcinoma (PDAC) accounts for more than 80% of pancreatic neoplasms [1, 23]. However, there are few studies on the prognostic value of GPC family genes in early-stage PDAC after pancreaticoduodenectomy despite the poor prognosis of this tumor type. In this study, we explored the relationship between GPC family genes expression and prognosis of PDAC patients.

## Methods

### Patient data

The RNA-sequencing dataset used in this study and the corresponding clinical data were acquired from The Cancer Genome Atlas (TCGA) (https://portal.gdc.cancer.gov/; accessed September 25, 2019), and *DESeq* was applied to normalize the initial material [24]. To increase reliability of data analysis, previously established inclusion and exclusion criteria were used [25]. The inclusion criteria were as follows: (i) survival information was complete; (ii) histology result was confirmed as PDAC; (iii) pathologic stage was I or II; (iv) pancreaticoduodenectomy was carried out on patients. PDAC patients with pathologic stage III or IV and those who underwent other surgical procedures were excluded from the study. According to the above criteria, 112 patients were included in the analysis. The clinical characteristics included in the analysis were age, sex, alcohol history, pathologic stage, histologic grade, radical resection, radiation therapy, targeted molecular therapy, overall survival (OS) time, and survival status. Dataset GSE62452 was downloaded from Gene Expression Omnibus (GEO) database to validate the prognostic value of survival-related genes (https://www.ncbi.nlm.nih.gov/geo/query/acc.cgi?acc=GSE62452; accessed October 5, 2020). Following the same criteria described above, we included 48 cases in this study.

### Analysis using public database

The expression status of GPC family genes in different normal tissues was analyzed by the Genotype-Tissue Expression (GTEx, https://www.gtexportal.org/, accessed October 9, 2019) website [26, 27]. The Gene Expression Profiling Interactive Analysis (GEPIA, http://gepia.cancer-pku.cn/, accessed October 9, 2019), an online tool containing 9736 tumors and 8587 normal samples from the TCGA and the GTEx projects, was used to show expression level of each gene in both tumor and normal tissues of PDAC [28]. The Database for Annotation, Visualization, and Integrated Discovery (DAVID) v6.8 (https://david.ncifcrf.gov/, accessed November 6, 2019) [29, 30] was chosen to carry out gene enrichment analysis containing Gene Ontology (GO) function analysis and Kyoto Encyclopedia of Genes and Genomes (KEGG) pathway analysis. The possible functioning pathways of the genes were also investigated by Biological Network Gene Ontology (BiNGO) in Cytoscape (version 3.7.1) [31].

### Survival analysis

Two groups of patients were set up based on 50% cutoff expression value of each gene both in TCGA database and GEO database. The relationship between OS and gene expression level as well as basic clinical characteristics was analyzed using Kaplan-Meier method with the log-rank test. Log-rank *P* < 0.05 was considered statistically significant. Multivariate Cox proportional hazards regression analysis was used to adjust for prognosis-significant factors. Hazard ratio (HR) and 95% confidence interval (CI) were considered to estimate the relative risk. Stratified analysis was carried out based on certain clinical characteristics of the patients for survival-related genes to explore their significance in prognosis. To understand the relationship between GPC genes and prognosis at a deeper level, joint effects survival analysis was taken into consideration. The survival-significant clinical characteristics, clinical factors usually related to prognosis of patients with malignant tumors clinically and prognosis-related genes were included to establish a prognosis nomogram. Better survival prediction could be made according to the total points.

### Genome-wide co-expression analysis

Genome-wide co-expression analysis of prognosis-related genes was performed to investigate their potential biological mechanisms based on TCGA database. A gene with Pearson correlation coefficient > 0.5 and *P* < 0.05 was considered as a co-expression gene. A co-expression network was built for each gene related to prognosis and its co-expressed genes using Cytoscape software (version 3.7.1) [32]. GO function analysis and KEGG pathway analysis of these genes were also completed using DAVID [29, 30].

### Gene set enrichment analysis

To understand the underlying mechanisms of GPC genes affecting prognosis, we used Gene Set Enrichment Analysis (GSEA, http://software.broadinstitute.org/gsea/index.jsp, November 6, 2019) [33, 34]. Databases c2 (c2.all.v7.0.symbols.gmt) and c6 (c6.all.v7.0.symbols.gmt) in the Molecular Signatures Database (MSigDB) [35] were used to search for possible pathways based on TCGA database. Enrichment results were considered statistically significant if the nominal *P*-value was < 0.05 and the false discovery rate (FDR) was < 0.25.

### Statistical analysis

Survival analysis was performed using Kaplan-Meier method with log-rank test. Univariate and multivariate survival analyses were performed with Cox proportional hazards regression model to calculate crude and adjusted HRs and 95% CIs. Survival curves were plotted using GraphPad Prism v.7.0 (GraphPad Software Inc., La Jolla, CA).

The unpaired *t* test was used to compare gene expression levels between normal and tumor tissues. The expression relationship of each GPC gene and its co-expressed genes was quantified by Pearson’s correlation coefficient. The correlation plot was constructed using Cytoscape software (version 3.7.1). All statistical analyses were performed using SPSS v.25.0 software (IBM, Chicago, IL, USA). A *P* value < 0.05 was considered statistically significant.

## Results

### Analysis using public database

**Fig. 1:**
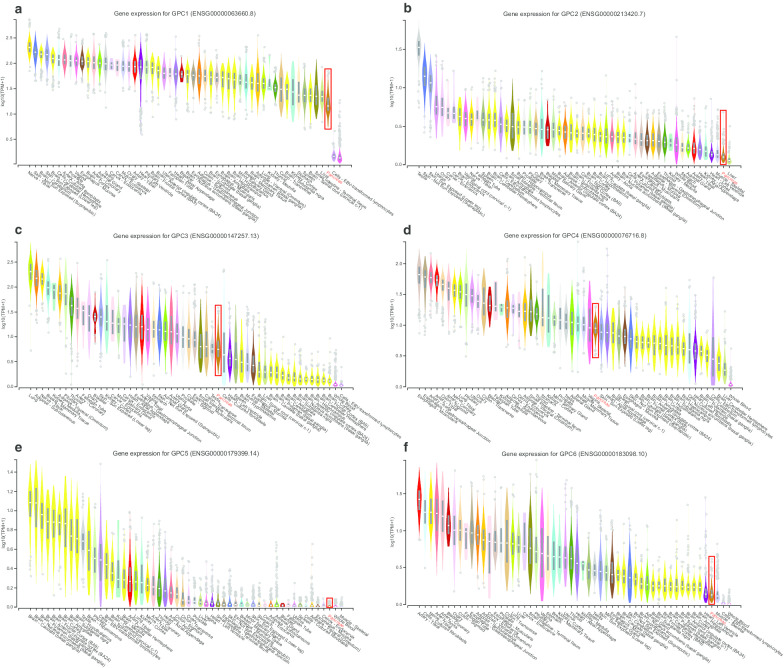
Gene expression levels of Glypican family genes in normal human organ tissues. **a**-**f** Gene expression levels of *Glypican1–6*, respectively

**Fig. 2:**
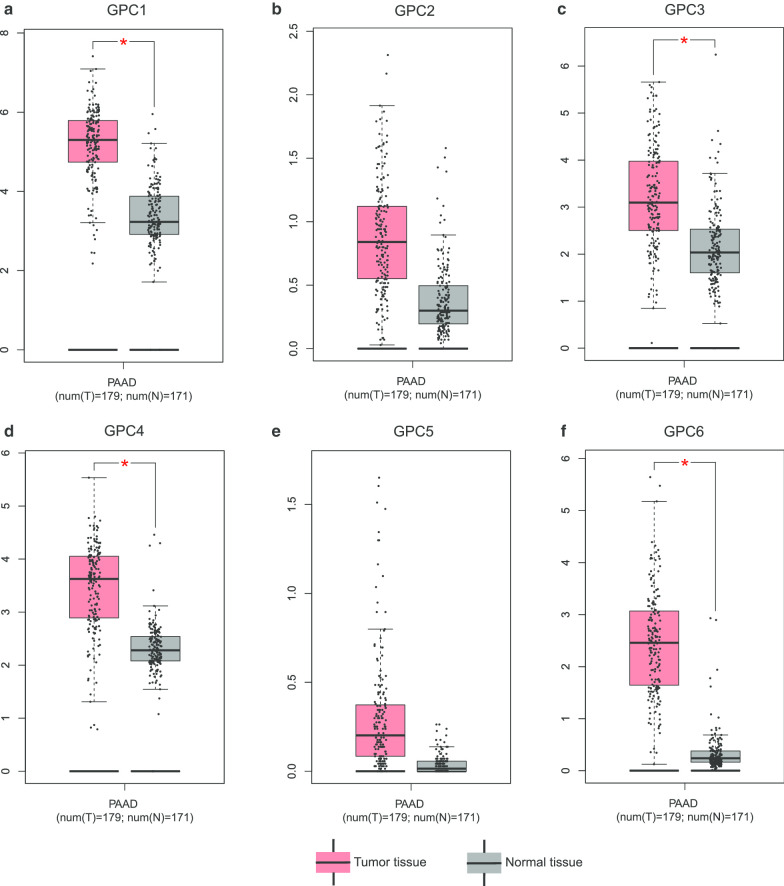
Gene level distribution of Glypican family genes in pancreatic ductal adenocarcinoma between tumor and normal tissues. **a**-**f** Gene level distribution of *Glypican1*–*6* in pancreatic ductal adenocarcinoma between tumor and normal tissues, respectively. Notes: *P < 0.05.

**Fig. 3:**
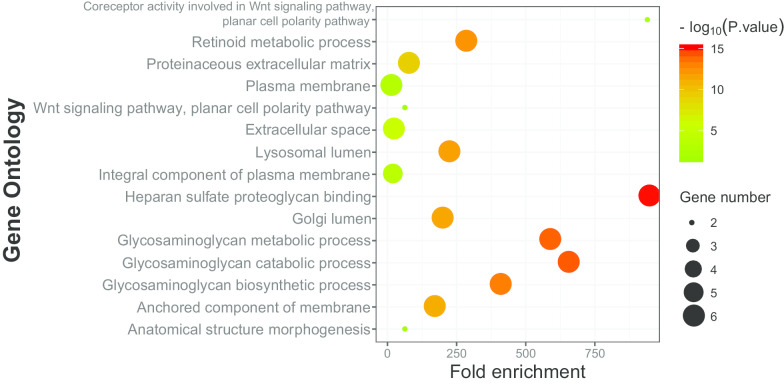
Function enrichment analysis of Gene Ontology for Glypican family genes completed by the Database for Annotation, Visualization, and Integrated Discovery

**Fig. 4:**
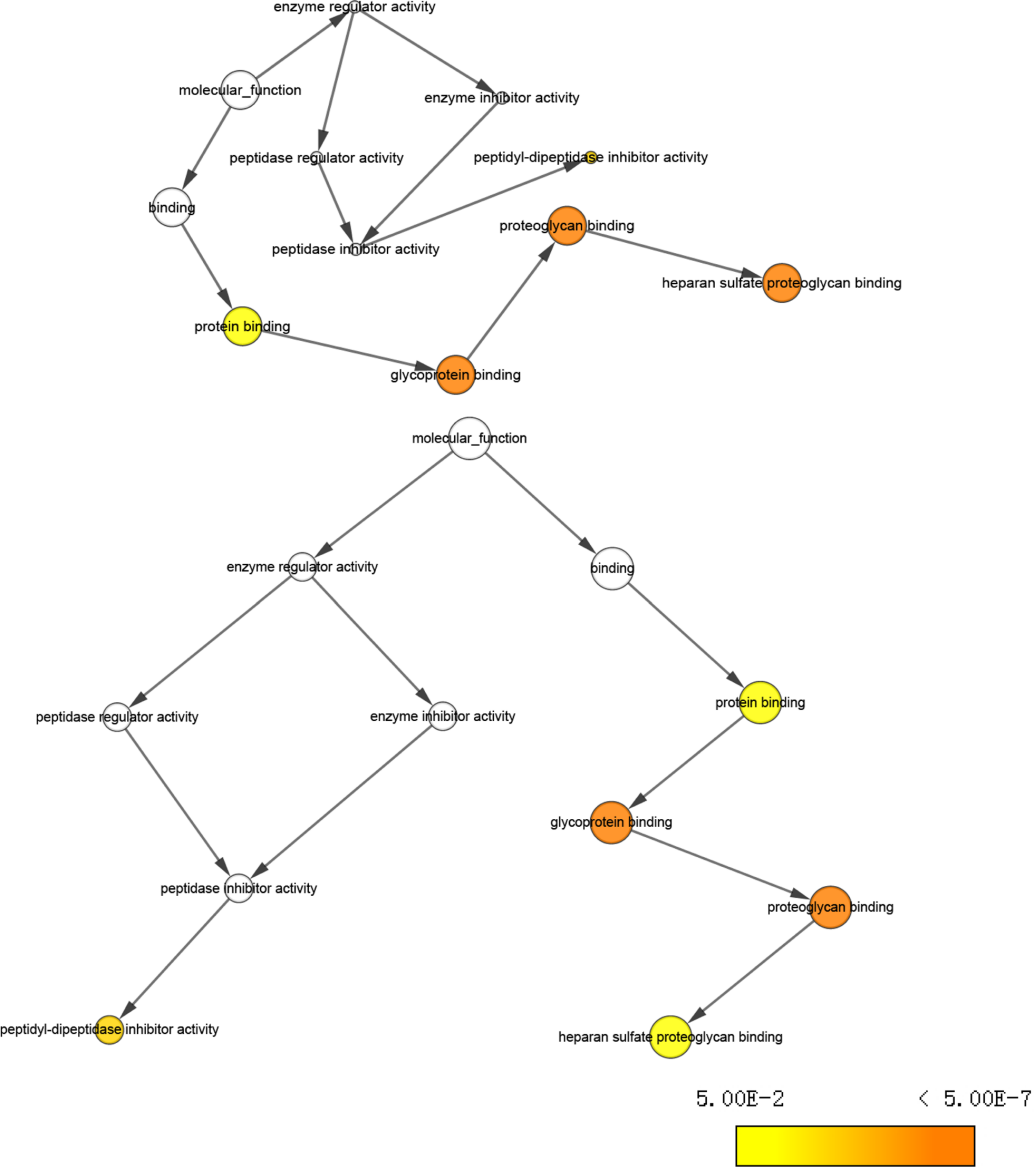
Functioning pathways of Glypican family genes carried out by Biological Network Gene Ontology in Cytoscape software

The expression status of GPC family genes in tissues derived from various normal human organs was analyzed using GTEx (Fig. 1). The expression level of GPC family genes was lower in human pancreas than in other organs. The results of GEPIA analysis showed that expression of *GPC1*, *GPC3*, *GPC4*, and *GPC6* was significantly higher in PDAC tumor tissues than in normal tissues (*P* < 0.05) (Fig. 2). GO functional enrichment analysis indicated that GPC family genes were mainly involved in composition of cell membrane, organelles and anchored components of the membrane, heparan sulfate proteoglycan binding, and glycosaminoglycan metabolic process (Fig. 3, Additional file 1: Table 1). The results of BiNGO analysis (Fig. 4) confirmed those of GO analysis.

### Survival analysis

**Fig. 5:**
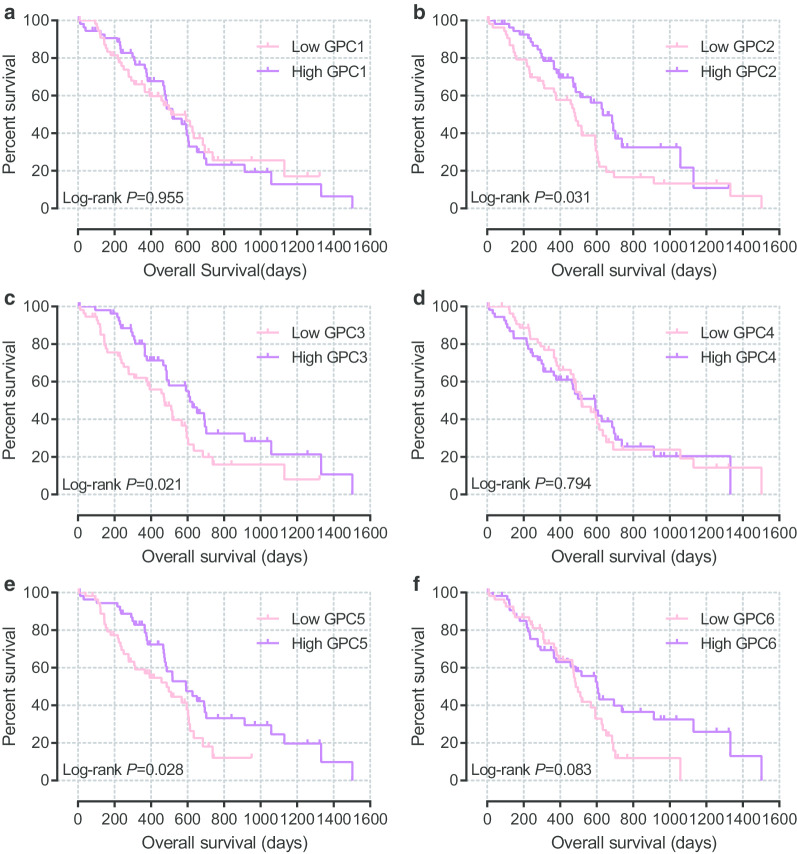
Kaplan-Meier survival curves of Glypican family genes for pancreatic ductal adenocarcinoma in The Cancer Genome Atlas database. **a**-**f** Kaplan-Meier survival curves of *Glypican1*–*6*, respectively

**Fig. 6:**
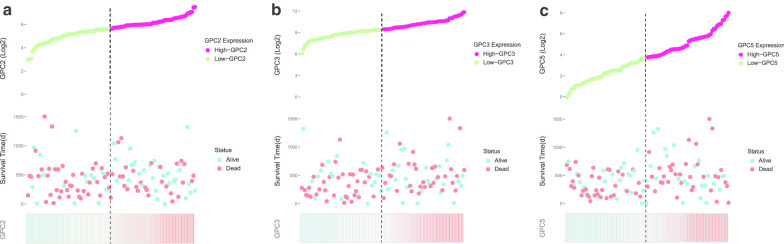
Prognostic models of *Glypican2*, *Glypican3* and *Glypican5* for pancreatic ductal adenocarcinoma in The Cancer Genome Atlas database. From top to bottom are expression values of these genes, survival status of patients and expression heatmaps of these genes at low and high expression levels. **a**-**c** Prognostic models of *Glypican2*, *Glypican3,* and *Glypican5*, respectively

The Kaplan-Meier method and log-rank test were used to investigate the association between basic clinical characteristics and OS in TCGA database. Additional file 2: Table 2 shows that histologic grade, extent of surgery, treatment with radiation and targeted molecular therapy were significant in OS. GPC family genes were divided into two groups based on expression level, and survival analysis was performed between the two groups. The results (Fig. 5a–f) demonstrated that expression of *GPC2*, *GPC3*, and *GPC5* was significantly associated with survival. The median survival time (MST) was significantly longer in patients with high expression of *GPC2*, *GPC3*, and *GPC5* than the low expression group (log-rank *P* = 0.031, 0.021, and 0.028, respectively; MST, 634 days vs. 481 days, 614 days vs. 473 days, and 593 days vs. 485 days, respectively, Fig. 5b, c, e and Fig. 6). After adjusting for survival-significant clinical parameters in a multivariate Cox proportional hazards regression model, *GPC2*, *GPC3*, and *GPC5* were still significantly associated with OS (Table 1) (adjusted *P* = 0.005, adjusted HR = 0.449, 95% CI = 0.258–0.782; adjusted *P* = 0.022, adjusted HR = 0.531, 95% CI = 0.309–0.914; and adjusted *P* = 0.020, adjusted HR = 0.525, 95% CI = 0.306–0.902, respectively). Results of stratified analysis for *GPC2*, *GPC3*, and *GPC5* are shown in Table 2. High expression of *GPC2* was significantly associated with better OS in patients who were male, were > 60 years old, had histologic grade G1 or G2, had R1 or Rx resection or whether received radiation therapy. *GPC3* expression was related to patients who were female, were > 60 years old, had histologic grade G1 or G2, or did not receive radiation or targeted molecular therapy. Moreover, *GPC5* could influence prognosis of patients who were ≤ 60 years old, had histologic grade G3 or G4, had R1 or Rx resection, or did not receive radiation or targeted molecular therapy.

**Table 1:** Prognostic value of Glypican family genes in The Cancer Genome Atlas database

Gene	No. of events (%)	MST (days)	Crude HR 95% CI	Crude *P* value	Adjusted HR 95% CI^a^	Adjusted *P* value^a^
*GPC1*						
Low	36/56(64.3%)	518	1		1	
High	33/56(58.9%)	511	0.986(0.610–1.594)	0.955	1.120 (0.661–1.896)	0.674
*GPC2*						
Low	41/56(73.2%)	481	1		1	
High	28/56(50.0%)	634	0.589(0.362–0.959)	0.031	0.449(0.258–0.782)	0.005
*GPC3*						
Low	38/56(67.9%)	473	1		1	
High	31/56(55.4%)	614	0.568(0.349–0.925)	0.021	0.531(0.309–0.914)	0.022
*GPC4*						
Low	35/56(62.5%)	517	1		1	
High	34/56(60.7%)	592	1.066(0.659–1.723)	0.794	1.252(0.744–2.105)	0.397
*GPC5*						
Low	35/56(62.5%)	485	1		1	
High	34/56(60.7%)	593	0.577(0.351–0.948)	0.028	0.525(0.306–0.902)	0.020
*GPC6*						
Low	37/56(66.1%)	485	1		1	
High	32/56(57.1%)	603	0.647(0.393–1.063)	0.083	0.891(0.501–1.585)	0.693

*Abbreviations: MST* median survival time, *HR* hazard ratio, *CI* confidence interval

^a^Adjusted for histologic grade, targeted molecular therapy, radiation therapy and radical resection

**Table 2:** Stratified analysis of Glypican genes in The Cancer Genome Atlas database

	*GPC2*				*GPC3*				*GPC5*			
	Low	High	Adjusted HR 95% CI	Adjusted P 95%CI ^a^	Low	High	Adjusted HR 95% CI	Adjusted P 95%CI ^a^	Low	High	Adjusted HR 95% CI	Adjusted P 95%CI ^a^
Age years)												
≤60	16	22	0.433 (0.119–1.569)	0.202	20	18	1.061 (0.238–4.729)	0.938	18	20	0.197 (0.041–0.951)	0.043
> 60	40	34	0.415 (0.218–0.790)	0.007	36	38	0.517 (0.280–0.954)	0.035	38	36	0.637 (0.342–1.185)	0.154
Sex												
Female	30	23	0.481 (0.189–1.221)	0.124	28	25	0.381 (0.160–0.909)	0.030	26	27	0.464 (0.200–1.076)	0.073
Male	26	33	0.302 (0.140–0.652)	0.002	28	31	0.880 (0.372–2.086)	0.772	30	29	0.477 (0.211–1.078)	0.075
Histologic grade												
G1 + G2	39	41	0.374 (0.178–0.787)	0.010	41	39	0.442 (0.222–0.880)	0.020	39	41	0.635 (0.322–1.254)	0.191
G3 + G4	17	15	0.495 (0.177–1.383)	0.180	15	17	0.644 (0.238–1.743)	0.386	17	15	0.303 (0.105–0.870)	0.026
Radical resection^b^												
R0	36	30	0.532 0.249–1.136)	0.103	29	37	0.589 0.293–1.184)	0.137	31	35	0.712 0.346–1.466)	0.357
R1 + Rx	20	24	0.378 0.162–0.880)	0.024	26	18	0.368 0.135–1.003)	0.051	25	19	0.305 0.120–0.770)	0.012
Radiation therapy^c^												
No	42	28	0.462 (0.241–0.885)	0.020	37	33	0.504 (0.271–0.938)	0.031	37	33	0.479 (0.254–0.904)	0.023
Yes	11	19	0.258 (0.068–0.977)	0.046	11	19	0.612 (0.166–2.260)	0.461	12	18	0.725 (0.210–2.498)	0.610
Targeted molecular therapy^d^												
No	15	14	0.453 (0.187–1.095)	0.079	19	10	0.346 (0.125–0.956)	0.041	16	13	0.397 (0.158–0.995)	0.049
Yes	38	35	0.477 (0.222–1.027)	0.059	30	43	0.668 (0.328–1.359)	0.265	33	40	0.571 (0.282–1.154)	0.118

*Abbreviations: HR* hazard ratio, *CI* confidence interval

^a^ Adjusted for histologic grade, targeted molecular therapy, radiation therapy and radical resection. ^b^ Information of radical resection was unavailable in 2 patients. ^c^ Information of radiation therapy was unavailable in 12 patients. ^d^ Information of targeted molecular therapy was unavailable in 10 patients

### Joint effects analysis

Based on the prognostic significance of each GPC family gene, we combined every two genes among *GPC2*, *GPC3*, and *GPC5* to investigate their significance in PDAC prognosis. The combination of *GPC2* and *GPC3* was associated with worse survival outcome in group 1 (MST = 278 days, adjusted *P* value < 0.001). The group of *GPC2* and *GPC5* was associated with the highest risk of death in group I (MST = 278 days, adjusted *P* value < 0.001) and the group combining *GPC3* and *GPC5* showed the poorest prognosis in group i (MST = 278 days, adjusted *P* value < 0.001).

**Fig. 7:**
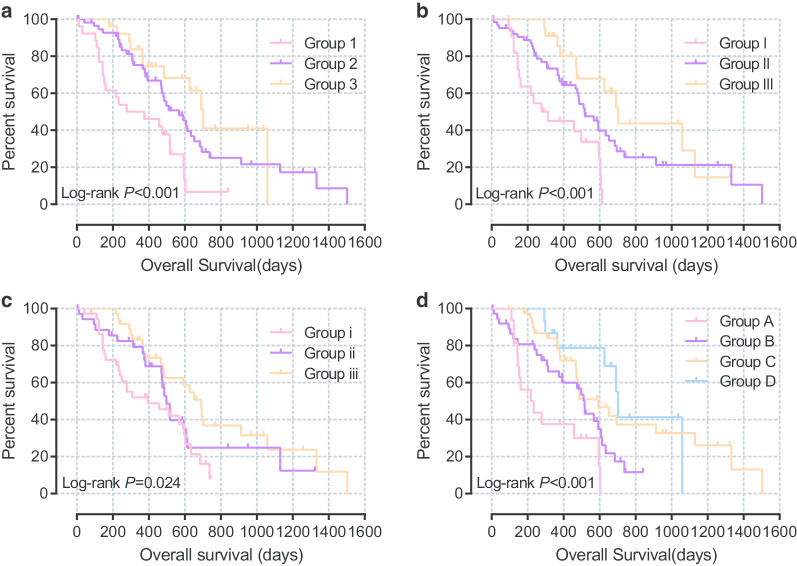
Survival curves of joint effects analysis of *Glypican2*, *Glypican3* and *Glypican5* in The Cancer Genome Atlas database. **a** Survival curve of *Glypican2* and *Glypican3*; **b** Survival curve of *Glypican2* and *Glypican5*; **c** Survival curve of *Glypican3* and *Glypican5*; **d** Survival curve of *Glypican2*, *Glypican3* and *Glypican5*

We also analyzed survival associated with the three genes simultaneously. Group A showed the worst in survival status (MST = 219 days, adjusted *P* value = 0.018), whereas the best survival was observed in group D (MST = 702 days, adjusted *P* value < 0.001). These data are shown in Table 3 and Fig. 7a–d showed the survival curves.

**Table 3:** Joint effects analysis of combination of Glypican genes in The Cancer Genome Atlas database

Group	No. of events	MST (days)	Crude HR 95% CI	Crude *P* value	Adjusted HR 95% CI^a^	Adjusted *P* value^a^
*GPC2* + *GPC3*						
1	21/26(80.8%)	278	1	0.001	1	< 0.001
2	37/60(61.7%)	568	0.441(0.252–0.772)	0.004	0.350(0.187–0.653)	0.001
3	11/26(42.3%)	702	0.285(0.135–0.598)	0.001	0.173(0.072–0.418)	< 0.001
*GPC2* + *GPC5*						
I	18/23(78.3%)	278	1	0.001	1	< 0.001
II	40/66(60.6%)	517	0.424(0.237–0.758)	0.004	0.283(0.145–0.554)	< 0.001
III	11/23(47.8%)	702	0.253(0.116–0.552)	0.001	0.141(0.057–0.353)	< 0.001
*GPC3* + *GPC5*						
i	26/38(68.4%)	393	1	0.028	1	0.018
ii	21/36(58.3%)	498	0.685(0.383–1.226)	0.203	0.575(0292–1.129)	0.108
iii	22/38(57.9%)	691	0.443(0.243–0.805)	0.008	0.394(0.206–0.756)	0.005
*GPC2* + *GPC3* + *GPC5*						
A	13/16(81.2%)	219	1	0.028	1	< 0.001
B	26/39(66.7%)	517	0.494(0.250–0.974)	0.042	0.446(0.212–0.938)	0.033
C	23/42(54.8%)	592	0.269(0.130–0.560)	< 0.001	0.176(0.076–0.406)	< 0.001
D	7/15(46.7%)	702	0.204(0.079–0.526)	0.001	0.135(0.045–0.403)	< 0.001

Group 1:low *GPC2* + low *GPC3*; Group 2:low *GPC2* + high *GPC3* or high *GPC2*+ low *GPC3*; Group 3: high *GPC2* + high *GPC3*

Group I:low *GPC2* + low *GPC5*; Group II:low *GPC2* + high *GPC5* or high *GPC2* + low *GPC5*; Group III: high *GPC2* + high *GPC5*

Group i:low *GPC3* + low *GPC5*; Group ii:low *GPC3* + high *GPC5* or high *GPC3* + low *GPC5*; Group iii: high *GPC3* + high *GPC5*

Group A:low *GPC2* + low *GPC3* + low *GPC5*; Group B: high *GPC2* + low *GPC3* + low *GPC5* or low *GPC2* + high *GPC3* + low *GPC5* or low *GPC2* + low *GPC3* + high *GPC5*; Group C:high *GPC2* + high *GPC3* + low *GPC5* or high *GPC2* + low *GPC3* + high *GPC5* or low *GPC2* + high *GPC3* + high *GPC5*; Group D:high *GPC2* + high *GPC3* + high *GPC5*

*Abbreviations: MST* median survival time, *HR* hazard ratio, *CI* confidence interval

^a^Adjusted for histologic grade, targeted molecular therapy, radiation therapy and radical resection

### Prognosis nomogram

**Fig. 8:**
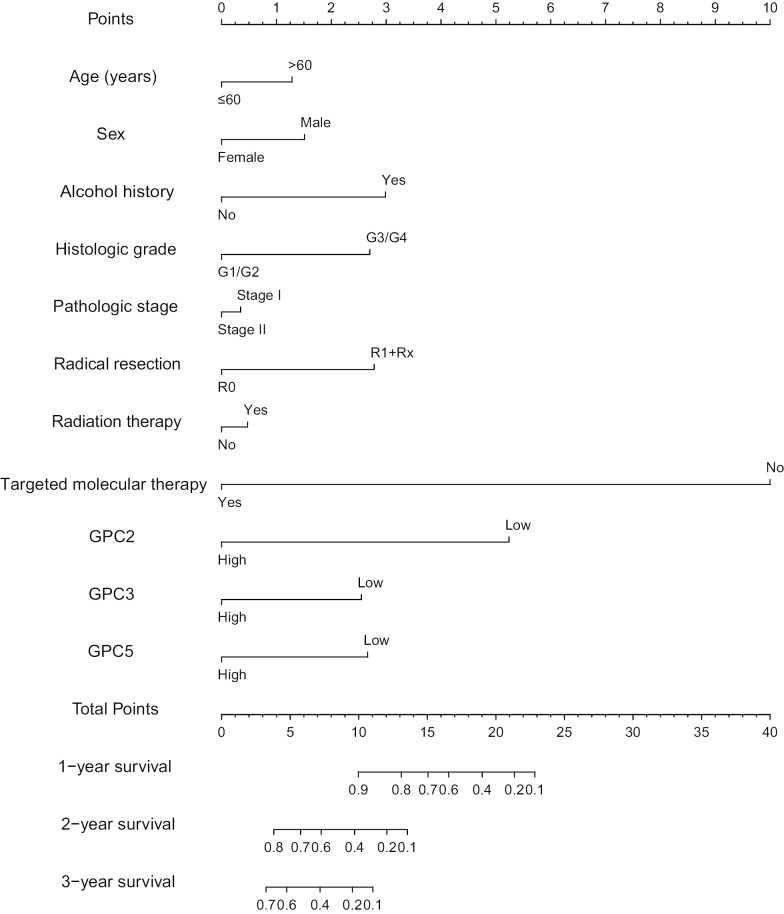
Prognosis nomogram for predicting 1-, 2- and 3- overall survival

Based on the status of each clinical parameter and expression levels of *GPC2*, *GPC3*, and *GPC5*, a score for each variable was calculated. The total score could be calculated to predict 1-, 2-, and 3- year survival probabilities. The nomogram (Fig. 8) indicated that *GPC2*, *GPC3*, and *GPC5* affected the prognosis of PDAC to different degrees.

### Validation dataset to demonstrate the prognostic value of survival-related genes

**Fig. 9:**
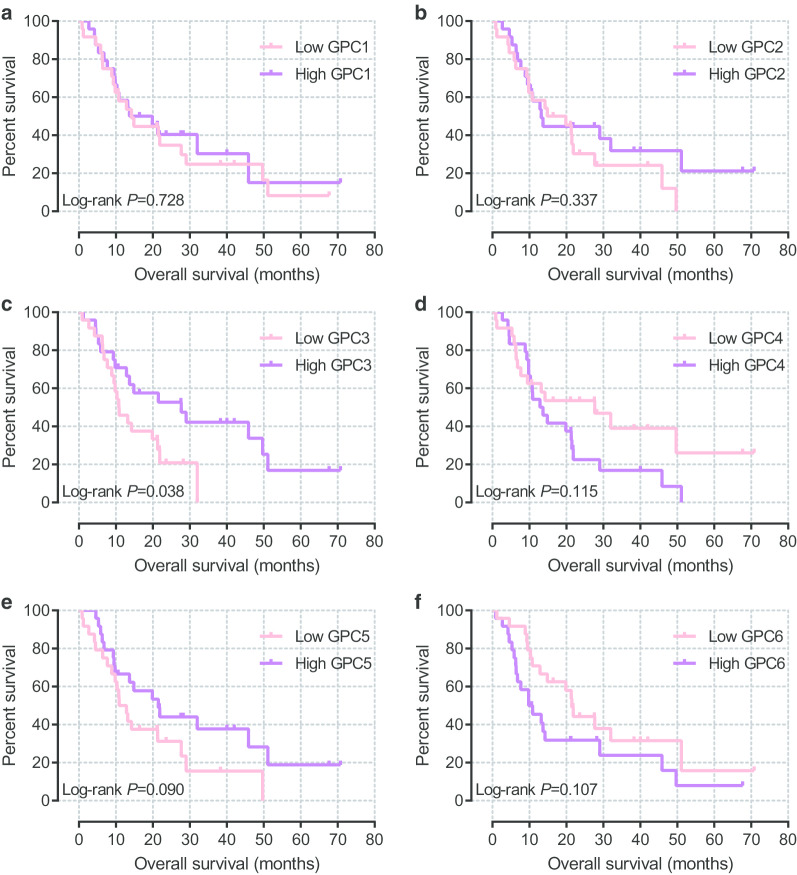
Kaplan-Meier survival curves of Glypican family genes for pancreatic ductal adenocarcinoma in Gene Expression Omnibus database. **a**-**f** Kaplan-Meier survival curves of *Glypican1*–*6*, respectively

To further understand the prognostic value of *GPC2*, *GPC3*, and *GPC5*, we acquired the GSE62452 dataset from GEO database. As shown in Additional file 3: Table 3, histologic grade was significantly associated with OS. GPC family genes were also divided into two groups by the median expression level of each gene and survival analysis between the two groups was carried out. Table 4 and Fig. 9a–f show that higher expression of *GPC3* was significantly related to better survival (log-rank *P* = 0.038) and higher expression of *GPC2* and *GPC5* was also related to better survival, though not significantly (log-rank *P* = 0.337 and 0.090, repectively). Multivariate Cox proportional hazards regression analysis adjusted for prognosis-related clinical characteristics showed that none of these genes was significantly correlated to overall survival (all adjusted *P* > 0.05).

**Table 4:** Prognostic value of Glypican family genes in Gene Expression Omnibus database

Gene	Samples (*n* = 48)	Crude HR 95% CI	Crude *P* value	Adjusted HR 95% CI^a^	Adjusted *P* value^a^
*GPC1*					
Low	24	1		1	
High	24	0.888(0.455–1.735)	0.728	0.675(0.330–1.383)	0.283
*GPC2*					
Low	24	1		1	
High	24	0.717(0.362–1.420)	0.337	0.852(0.418–1.738)	0.660
*GPC3*					
Low	24	1		1	
High	24	0.468(0.225–0.973)	0.038	0.556(0.259–1.197)	0.134
*GPC4*					
Low	24	1			
High	24	1.722(0.869–3.413)	0.115	1.616(0.812–3.216)	0.172
*GPC5*					
Low	24	1		1	
High	24	0.556(0.279–1.106)	0.090	0.600(0.298–1.206)	0.151
*GPC6*					
Low	24	1		1	
High	24	1.727(0.882–3.381)	0.107	1.554(0.783–3.083)	0.207

*Abbreviations: HR* hazard ratio, *CI* confidence interval

^a^Adjusted for histologic grade

### Genome-wide co-expression analysis of *GPC2*, *GPC3* and *GPC5* in PDAC

**Fig. 10:**
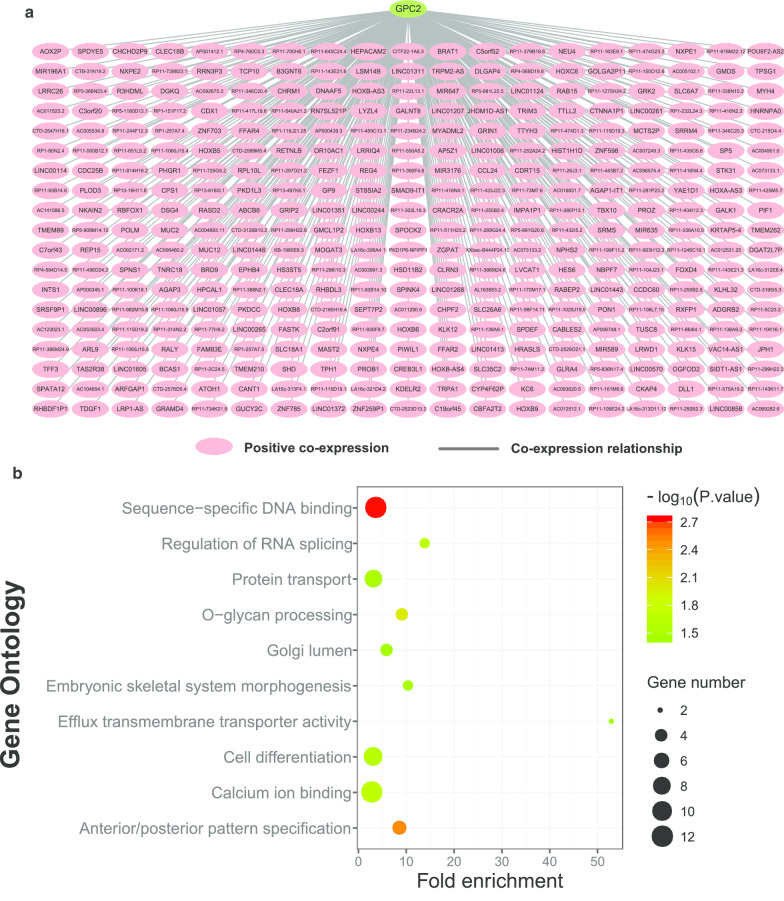
**a** Correlation network for *Glypican2* and its co-expression genes in The Cancer Genome Atlas database. The pink nodes are genes correlated positively. **b** Function enrichment analysis of Gene Ontology for *Glypican2* and its co-expression genes

Genome-wide co-expression analysis was performed for each of these genes to investigate their related functional pathways through TCGA database. For *GPC2* and its co-expressed genes, a correlation network was established as shown in Fig. 10a (Additional file 4: Table 4). GO analysis indicated that *GPC2* and its co-expressed genes functioned mainly in sequence-specific DNA binding, protein transport, cell differentiation, and anterior/posterior pattern specification (Fig. 10b, Additional file 5: Table 5).

**Fig. 11:**
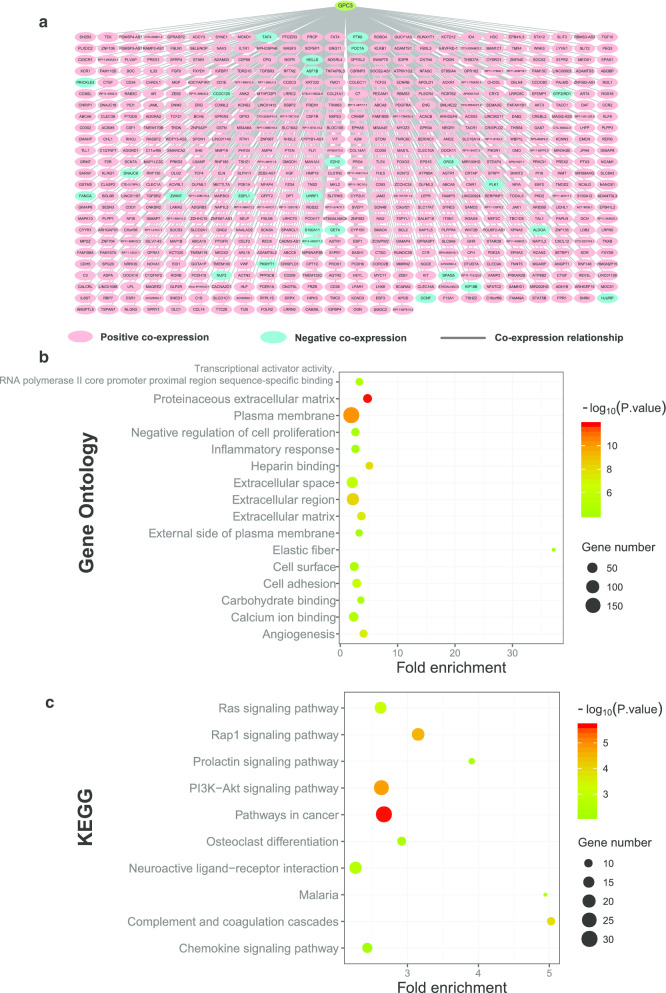
**a** Correlation network for *Glypican3* and its co-expression genes in The Cancer Genome Atlas database. The pink nodes are genes correlated positively and the blue nodes are genes correlated negatively. **b** Function enrichment analysis of Gene Ontology for *Glypican3* and its co-expression genes. **c** Function enrichment analysis of Kyoto Encyclopedia of Genes and Genomes for *Glypican3* and its co-expression genes

The correlation network for *GPC3* and its co-expressed genes (Fig. 11a, Additional file 6: Table 6) identified 511 positively co-expressed genes and 25 negatively co-expressed genes. GO analysis of these genes indicated that they were enriched in cell adhesion, angiogenesis, and inflammatory response (Fig. 11b, Additional file 7: Table 7). And KEGG analysis indicated that these genes were related to several biological processes, mainly in Ras, Rap1, PI3K-Akt, and chemokine signaling pathways (Fig. 11c, Additional file 8: Table 8).

**Fig. 12:**
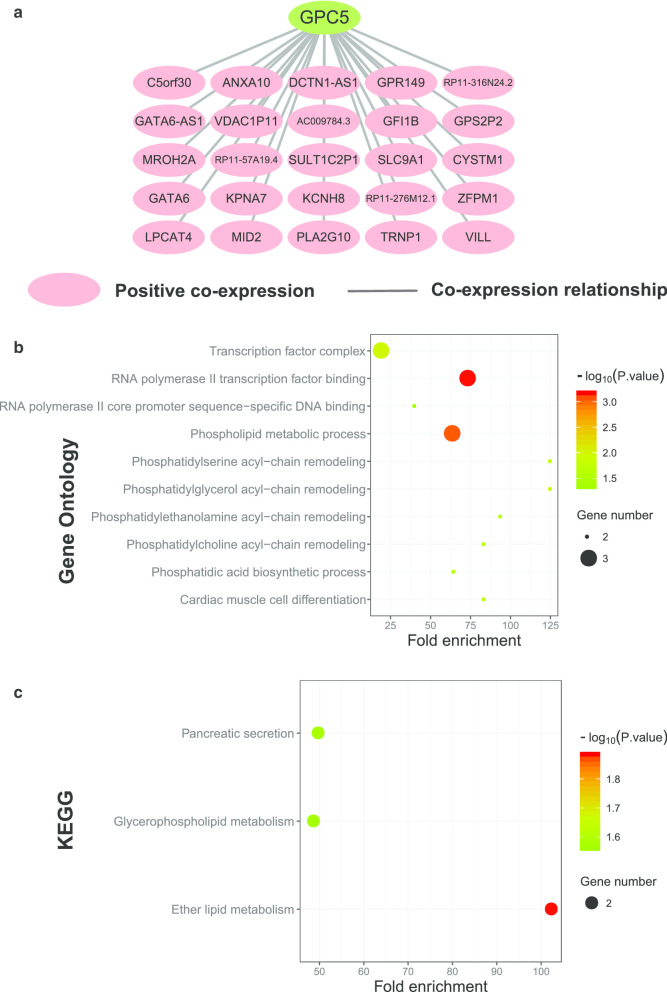
**a** Correlation network for *Glypican5* and its co-expression genes in The Cancer Genome Atlas database. The pink nodes are genes correlated positively. **b** Function enrichment analysis of Gene Ontology for *Glypican5* and its co-expression genes. **c** Function enrichment analysis of Kyoto Encyclopedia of Genes and Genomes for *Glypican5* and its co-expression genes

The correlation network for *GPC5* and its co-expressed genes was shown in Fig. 12a and Additional file 9: Table 9. The results of GO analysis showed that these genes were associated with transcription factor complex and phospholipid metabolic process (Fig. 12b, Additional file 10: Table 10). KEGG analysis showed that these genes were involved in pancreatic secretion and glycerophospholipid metabolism (Fig. 12c, Additional file 11: Table 11).

### Gene set enrichment analysis

**Fig. 13:**
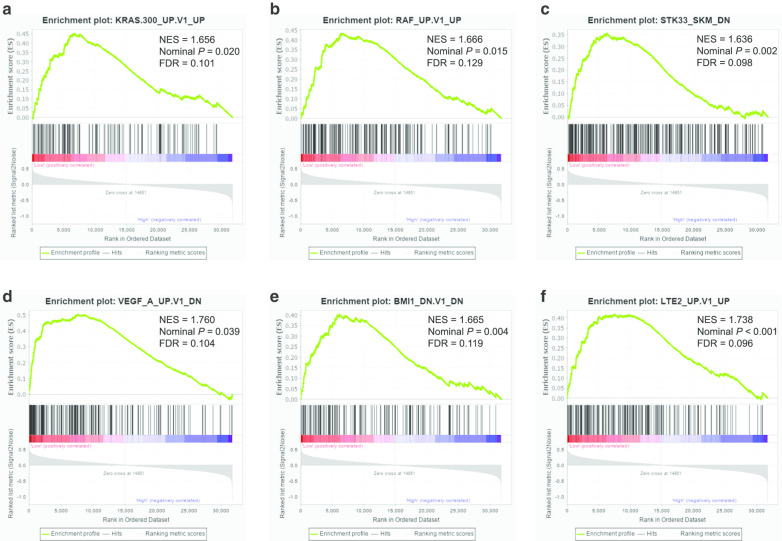
Gene Set Enrichment Analysis (GSEA) results of *Glypican2* in The Cancer Genome Atlas database. **a**-**f** GSEA results of c6 reference for the group of low *Glypican2* expression. NES, normalized enrichment score; FDR, false discovery rate

**Fig. 14:**
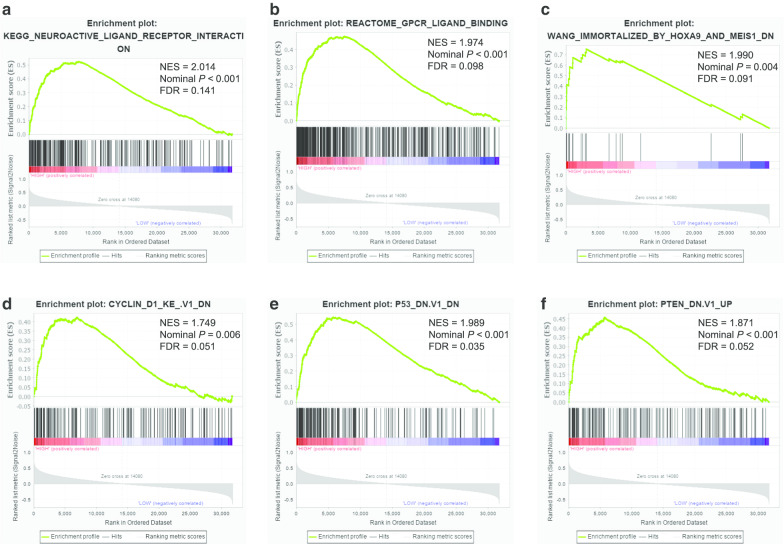
Gene Set Enrichment Analysis (GSEA) results of *Glypican3* in The Cancer Genome Atlas database. **a**-**c** GSEA results of c2 reference for the group of high *Glypican3* expression; **d**-**f** GSEA results of c6 reference for the group of high *Glypican3* expression. NES, normalized enrichment score; FDR, false discovery rate

**Fig. 15:**
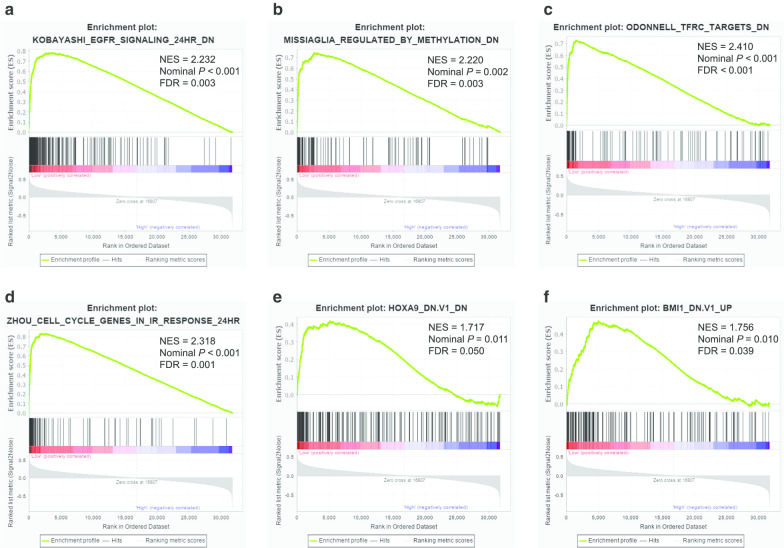
Gene Set Enrichment Analysis (GSEA) results of *Glypican5* in The Cancer Genome Atlas database. **a**-**d** GSEA results of c2 reference for the group of low *Glypican 5* expression; **e**-**f** GSEA results of c6 reference for the group of low *GPC5* expression. NES, normalized enrichment score; FDR, false discovery rate

GSEA was carried out to explore possible mechanisms of GPC family genes affecting prognosis of PDAC patients through TCGA database. The results of c6 reference indicated that low *GPC2* expression was closely related to oncogenic signatures such as KRAS, RAF1, STK33, and VEGFA (Fig. 13a–f; Additional file 12: Table 12). GSEA results of c2 enrichment showed that high *GPC3* expression was associated with neuroactive ligand receptor interaction and GPCR ligand binding (Fig. 14a–c; Additional file 13: Table 13), and c6 enrichment suggested that high *GPC3* expression was correlated to cyclin D1, p53, and PTEN (Fig. 13d–f; Additional file 14: Table 14). For *GPC5*, c2 reference indicated that low expression of *GPC5* was related to the EGFR pathway, gene methylation status, TFRC1, and the cell cycle (Fig. 15a–d; Additional file 15: Table 15) and c6 reference indicated that low *GPC5* expression was related to HOXA9 and BMI1 (Fig. 15e–f; Additional file 16: Table 16).

## Discussion

In this research, we studied the relationship between GPC family gene expression and prognosis of early-stage PDAC patients after pancreaticoduodenectomy both in TCGA database and GEO database. We concluded that high expression of *GPC2*, *GPC3*, and *GPC5* was significantly related to favorable prognosis in TCGA database, suggesting the value of these genes as biomarkers for predicting the prognosis of PDAC patients. Moreover, combination of the three genes showed a better predictive value for PDAC prognosis.

GPC family genes may contribute to the malignant behaviors of tumors and they are closely related to the development and prognosis of various cancers. Li et al. demonstrated that GPC1 is enriched in exosomes produced by colorectal cancer cells HT-29 and HCT-116, and increased expression level of miR-96-5p and miR-149 can restrain both GPC1 expression and cell proliferation of the tumor, suggesting that GPC1 can be used as a marker for diagnosis and therapy of colorectal cancer [36]. It is reported that *GPC2* could promote the proliferation of neuroblastoma cells as a result of MYCN binding to a motif of the promoter of *GPC2* and gain of chromosome 7q [37]. *GPC2* can also be used an effective prognostic indicator for prostate cancer and neuroblastoma [37–39]. *GPC3* blocks the cell cycle in renal cancer cells 786-O and ACHN at G1 phase [40]. Overexpression of *GPC3* reduces progression and metastasis of breast cancer cells LM3 through targeting canonical Wnt pathway [41]. The *GPC5* rs2352028 variant and lower expression of this gene may contribute to increased risk of lung cancer [42, 43]. Sun et al. have shown that *GPC5* regulates epithelial–mesenchymal transition to reduce invasion of prostate cancer cells [44]. Its expression can serve as a prognostic indicator in a cohort of prostate cancer patients in China [45]. In this study, we demonstrated the relationship between OS and expression levels of *GPC2*, *GPC3*, and *GPC5*. Combined with results of GEPIA, it demonstrates their roles as tumor suppressor genes in PDAC.

To explore potential mechanisms of GPC genes affecting prognosis, we conducted GSEA and genome-wide co-expression analyses. The results showed that *GPC2* was associated with sequence-specific DNA binding, protein transport, cell differentiation and oncogenic signatures (KRAS, RAF, STK33, and VEGFA). In pancreatic cancer, mutation of *TP53* at codon 249 can alter the structure of p53, thus affecting its binding to a specific region of DNA and enhancing the risk of cancer [46, 47]. A study showed that GDF11 regulates the biological behaviors of pancreatic cancer cells to influence their differentiation and high expression of GDF11 is associated with favorable OS in pancreatic cancer [48]. RAF1 accelerates migration and invasion of pancreatic cancer and disorders of the RAF1 pathway are related to worse prognosis in pancreatic cancer patients [49, 50]. Moreover, microRNA-216a may downregulate RAF1 in pancreatic cancer and increase cell apoptosis [51]. VEGFA expression can increase as a result of the long non-coding RNA (lncRNA) 00511 in PDAC, which finally promotes tumor progression. The expression level of lnc00511 can be used as an indicator of prognosis in PDAC [52].

*GPC3* is related to cell adhesion, angiogenesis, inflammatory response, signaling pathways like Ras, Rap1, PI3K-Akt, chemokine, GPCR, and signatures like cyclin D1, p53, PTEN. For pancreatic cancer patients, the degree of inflammatory response can be measured by serum lactate dehydrogenase level and it is associated with the outcome of patients [53]. Angiogenesis is dysregulated in PDAC, and it contributes to proliferation and deterioration of the tumor, making survival of patients worse [54, 55]. Certain mutations of *KRAS* are associated with the response to drugs in PDAC cells [56]. In PDAC associated with the *KRAS* mutation, decitabine therapy inhibits tumor growth [57]. ARF6 is reported to be in close relationship with the Ras pathway and its overexpression is related to unfavorable prognosis of PDAC patients [58]. PTEN plays a role in pancreatic cancer growth. The function of PTEN is regulated by HNF1A and finally affects the survival of pancreatic cancer patients [59, 60].

*GPC5* is involved in the transcription factor complex TFRC1, oncogenic signatures HOXA9 and BMI1, gene methylation, phospholipid metabolic process, glycerophospholipid metabolism, cell cycle, and the EGFR pathway. In pancreatic cancer, the transcription factor hif- 2α can speed up metabolism and promote tumor proliferation and high level of hif- 2α correlates with worse OS [61, 62]. The methylation status of *GRAP2*, *ICAM3*, *A2ML1*, *MUC1,* and *MUC4* can influence the expression of these genes, which is associated with survival of pancreatic cancer [63, 64]. Phosphatidylserine is related to apoptosis of pancreatic cancer cells with the involvement of microparticles [65]. Stimuli such as oxidative stress can make phosphatidylserine appear outside on the pancreatic cancer cell membrane, finally leading to dysregulation of factors and cells such as VEGF and macrophages, making prognosis of patients unfavorable [66–68]. The EGFR pathway contributes to pancreatic cancer growth and accelerates invasion of the cancer as a result of lnc00976 overexpression, which can deteriorate the outcome of patients [69, 70].

The present study had several limitations. First, clinical data acquired from TCGA and GEO databases did not include all the relevant information, and there may be some factors that needed to be adjusted. Second, because the study included PDAC patients who underwent pancreaticoduodenectomy, the sample size was relatively small. Third, the results of genome-wide analysis and GSEA analysis were based on online databases to predict potential processes influencing prognosis, and further studies at molecular and genomic levels are necessary to confirm the results.

Despite these limitations, we identified *GPC2*, *GPC3*, and *GPC5* as biomarkers for prognosis of PDAC patients and showed that joint effects analysis was more effective for prediction of prognosis. We also explored possible mechanisms of survival-significant genes affecting PDAC prognosis through genome-wide analysis and GSEA analysis. These results could all improve prognostic prediction for PDAC and provide information valuable for the management of PDAC patients and making better clinical decisions in this population.

## Conclusions

We identified *GPC2*, *GPC3*, and *GPC5* as potential prognostic indicators for PDAC patients and showed that combination of these genes was more effective for prognosis prediction. Possible mechanisms of *GPC2* influencing prognosis may involve sequence-specific DNA binding, protein transport, cell differentiation and oncogenic signatures (KRAS, RAF, STK33, and VEGFA). *GPC3* may be related to cell adhesion, angiogenesis, inflammatory response, signaling pathways such as Ras, Rap1, PI3K-Akt, chemokine, and GPCR, and signatures including cyclin D1, p53, and PTEN. *GPC5* may be involved in the transcription factor complex TFRC1, the oncogenic signatures HOXA9 and BMI1, gene methylation, the phospholipid metabolic process, glycerophospholipid metabolism, cell cycle, and the EGFR pathway.

## Supplementary information

**Additional file 1: Table 1.** Gene Ontology terms of Glypican family genes.

**Additional file 2: Table 2.** Basic characteristics of pancreatic ductal adenocarcinoma patients in The Cancer Genome Atlas database.

**Additional file 3: Table 3.** Basic characteristics of PDAC patients in Gene Expression Omnibus database.

**Additional file 4: Table 4.** Genome-wide co-expression genes of *Glypican2* in pancreatic ductal adenocarcinoma in The Cancer Genome Atlas database.

**Additional file 5: Table 5.** Gene Ontology terms of *Glypican2* and its co-expression genes in The Cancer Genome Atlas database.

**Additional file 6: Table 6.** Genome-wide co-expression genes of *Glypican3* in pancreatic ductal adenocarcinoma in The Cancer Genome Atlas database.

**Additional file 7: Table 7.** Gene Ontology terms of *Glypican3* and its co-expression genes in The Cancer Genome Atlas database.

**Additional file 8: Table 8.** Kyoto Encyclopedia of Genes and Genomes pathways of *Glypican3* and its co-expression genes in The Cancer Genome Atlas database.

**Additional file 9: Table 9.** Genome-wide co-expression genes of *Glypican5* in pancreatic ductal adenocarcinoma in The Cancer Genome Atlas database.

**Additional file 10: Table 10.** Gene Ontology terms of *Glypican5* and its co-expression genes in The Cancer Genome Atlas database.

**Additional file 11: Table 11.** Kyoto Encyclopedia of Genes and Genomes pathways of *Glypican5* and its co-expression genes in The Cancer Genome Atlas database.

**Additional file 12: Table 12.** Gene Set Enrichment Analysis results of c6 enrichment for low *Glypican2* expression in The Cancer Genome Atlas database.

**Additional file 13: Table 13.** Gene Set Enrichment Analysis results of c2 enrichment for high *Glypican3* expression in The Cancer Genome Atlas database.

**Additional file 14: Table 14.** Gene Set Enrichment Analysis results of c6 enrichment for high *Glypican3* expression in The Cancer Genome Atlas database.

**Additional file 15: Table 15.** Gene Set Enrichment Analysis results of c2 enrichment for low *Glypican5* expression in The Cancer Genome Atlas database.

**Additional file 16: Table 16.** Gene Set Enrichment Analysis results of c6 enrichment for low *Glypican5* expression in The Cancer Genome Atlas database.

## Data Availability

Datasets generated and analyzed during the current study are available from The Cancer Genome Atlas, https://portal.gdc.cancer.gov and Gene Expression Omnibus, https://www.ncbi.nlm.nih.gov/geo/query/acc.cgi?acc=GSE62452.
