# Supplementary material for: Prognostic value of Glypican family genes in early-stage pancreatic ductal adenocarcinoma after pancreaticoduodenectomy and possible mechanisms
Source: BMC Gastroenterol. 2020 Dec 10;20:415. doi: 10.1186/s12876-020-01560-0 (PMC7731467; doi:10.1186/s12876-020-01560-0)
Supplement: Supplementary file 2 — Additional file 2: Table 2. Basic characteristics of pancreatic ductal adenocarcinoma patients in The Cancer Genome Atlas database. [file 12876_2020_1560_MOESM2_ESM.docx]

Supplementary Table 2 Basic characteristics of pancreatic ductal adenocarcinoma patients in The Cancer Genome Atlas database.

| Variables | No. of events (%) | MST (days) | Crude HR  95% CI | Log-rank P-value |
| --- | --- | --- | --- | --- |
| Age(years) |  |  |  |  |
| ≤60 | 20(52.6%) | 593 | 1 |  |
| >60 | 49(66.2%) | 485 | 1.636(0.962-2.780) | 0.066 |
| Sex |  |  |  |  |
| Female | 36(67.9%) | 511 | 1 |  |
| Male | 33(55.9%) | 592 | 0.855(0.529-1.382) | 0.523 |
| Alcohol history^a^ |  |  |  |  |
| No | 25(58.1%) | 592 | 1 |  |
| Yes | 38(62.3%) | 511 | 1.276(0.765-2.128) | 0.349 |
| Pathologic stage |  |  |  |  |
| Stage Ⅰ | 4(50%) | 236 | 1 |  |
| Stage Ⅱ | 65(62.5%) | 518 | 1.038(0.375- 2.872) | 0.943 |
| Histologic grade |  |  |  |  |
| G1+G2 | 45(56.2%) | 596 | 1 |  |
| G3+G4 | 24(75.0%) | 470 | 1.919(1.156-3.185) | 0.010 |
| Radical resection^b^ |  |  |  |  |
| R0 | 39(59.1%) | 603 | 1 |  |
| R1+Rx | 29(65.9%) | 381 | 1.945(1.174-3.223) | 0.009 |
| Radiation therapy^c^ |  |  |  |  |
| No | 48(68.6%) | 473 | 1 |  |
| Yes | 15(50.0%) | 691 | 0.527(0.293-0.947) | 0.029 |
| Targeted molecular therapy^d^ |  |  |  |  |
| No | 24(82.8%) | 224 | 1 |  |
| Yes | 41(56.2%) | 634 | 0.168(0.095-0.296) | <0.001 |

Notes: a Information of alcohol history was unavailable in 8 patients. b Information of radical resection was unavailable in 2 patients. c Information of radiation therapy was unavailable in 12 patients. d Information of targeted molecular therapy was unavailable in 10 patients.

Abbreviations: MST, median survival time; HR, hazard ratio; CI, confidence interval.
