# Supplementary material for: Prognostic value of Glypican family genes in early-stage pancreatic ductal adenocarcinoma after pancreaticoduodenectomy and possible mechanisms
Source: BMC Gastroenterol. 2020 Dec 10;20:415. doi: 10.1186/s12876-020-01560-0 (PMC7731467; doi:10.1186/s12876-020-01560-0)
Supplement: Supplementary file 3 — Additional file 3: Table 3. Basic characteristics of PDAC patients in Gene Expression Omnibus database. [file 12876_2020_1560_MOESM3_ESM.docx]

Supplementary Table 3 Basic characteristics of pancreatic ductal adenocarcinoma patients in Gene Expression Omnibus database.

| Variables | No. of events (%) | MST (months) | Crude HR  95% CI | Log-rank P-value |
| --- | --- | --- | --- | --- |
| Pathologic stage |  |  |  |  |
| Stage Ⅰ | 0(0) | 10 | 1 |  |
| Stage Ⅱ | 31(70.5) | 15 | 0.681(0.237-1.958) | 0.474 |
| Histologic grade |  |  |  |  |
| G1+G2 | 17(65.4) | 22 | 1 |  |
| G3+G4 | 18(81.8) | 13 | 2.038(1.008-4.120) | 0.043 |

Abbreviations: MST, median survival time; HR, hazard ratio; CI, confidence interval.
